# Amplifying Orbital Angular Momentum Modes in Ring-Core Erbium-Doped Fiber

**DOI:** 10.34133/2020/7623751

**Published:** 2020-02-20

**Authors:** Jun Liu, Shi Chen, Hongya Wang, Shuang Zheng, Long Zhu, Andong Wang, Lulu Wang, Cheng Du, Jian Wang

**Affiliations:** ^1^Wuhan National Laboratory for Optoelectronics and School of Optical and Electronic Information, Huazhong University of Science and Technology, Wuhan, 430074 Hubei, China; ^2^Fiberhome Telecommunication Technologies Co. Ltd., Wuhan 430074, China

## Abstract

Lots of research efforts have been devoted to increase the transmission capacity in optical communications using orbital angular momentum (OAM) multiplexing. To enable long-haul OAM mode transmission, an in-line OAM fiber amplifier is desired. A ring-core fiber (RCF) is considered to be a preferable design for stable OAM mode propagation in the fiber. Here, we demonstrate an OAM fiber amplifier based on a fabricated ring-core erbium-doped fiber (RC-EDF). We characterize the performance of the RC-EDF-assisted OAM fiber amplifier and demonstrate its use in OAM multiplexing communications with OAM modes carrying quadrature phase-shift keying (QPSK) and quadrature amplitude modulation (QAM) signals. The amplification of two OAM modes over four wavelengths is demonstrated in a data-carrying OAM-division multiplexing and wavelength-division multiplexing system. The obtained results show favorable performance of the RC-EDF-assisted OAM fiber amplifier. These demonstrations may open up new perspectives for long-haul transmission in capacity scaling fiber-optic communications employing OAM modes.

## 1. Introduction

Light beams carrying orbital angular momentum (OAM) are characterized by a helical phase front of exp(*ilθ*) [1], where *l* is the topological charge and *θ* is related to the azimuthal angle. The handedness and twisting rate of the helical phase front are described by the sign and absolute value of the topological charge *l*, respectively, which is an unlimited value in principle. Due to the helical phase structure, an OAM beam usually has a doughnut intensity profile with a phase singularity at the beam center. Because of these features, OAM beams have been widely used in optical manipulation, optical tweezers, microscopy and imaging, metrology, optical vortex knots, astronomy, and quantum information processing [2–14]. In the past few decades, optical communications employing OAM beams have also attracted increasing attention from researchers [15–21]. Similar to other mode bases in free space or in fiber [22–24], OAM beams with helical phase structure are another mode base related to the spatial domain. Thus, OAM-division multiplexing (OAM-DM), as a new multiplexing technique of space-division multiplexing (SDM), provides an alternative potential to enable the continuous increase of transmission capacity and spectral efficiency [15, 16, 25–31]. Ring-core fiber (RCF) with an annual refractive index profile has been proven to be a preferable design for stable propagation of OAM modes in fibers since being first demonstrated in 2009 [32]. The index profile of RCF is compatible with the donut-shaped OAM fields which is beneficial to suppress the unwanted radially higher-order modes. This simplifies the multiplexing and demultiplexing of the OAM modes and prevents crossing between effective indices of modes belonging to different families, allowing good mode separation. In addition, comparing to a mode-division multiplexing (MDM) system using multimode fibers (MMFs), fiber-optic OAM multiplexing communications employing RCFs can simplify or even avoid the complex multiple-input multiple-output (MIMO) digital signal processing (DSP) traditionally used to mitigate the mode crosstalk [33–35]. In the last decade, optimized design of RCFs has led to a significant increase in the number of OAM modes and the length of RCFs in fiber-optic OAM multiplexing communication systems [33–35]. However, the transmission distances are still very short for practical applications and cannot be compared with the widely used commercial single-mode fiber (SMF) optical communication systems. One of the limitations is the lack of OAM amplifiers. Among various types of amplifiers, fiber-based optical amplifiers are preferable for their full compatibility with fiber-optic communications. Hence, an OAM fiber amplifier is also highly desired, and its use in a practical data-carrying OAM multiplexing communication system is of great importance. Furthermore, the theoretical analyses show that erbium-doped fiber (EDF) with a ring-core profile features larger mode gain and smaller differential modal gain (DMG) [36]. In this scenario, a laudable goal would be to implement an OAM fiber amplifier based on the ring-core EDF (RC-EDF) with an annual refractive index profile.

In this paper, we propose and demonstrate an OAM fiber amplifier based on a fabricated RC-EDF. Two OAM modes (OAM_+1_ and OAM_−1_) each carrying a 10-Gbaud quadrature phase-shift keying (QPSK) or quadrature amplitude modulation (QAM) signal are multiplexed together and amplified by the RC-EDF-assisted OAM fiber amplifier. In addition, the compatibility of the RC-EDF-assisted OAM fiber amplifier with a wavelength-division multiplexing (WDM) system is evaluated by amplifying two OAM modes carrying 10-Gbaud QPSK signals over four wavelengths. Finally, we characterize in detail the performance of a single OAM mode carrying a 10-Gbaud QPSK/16-QAM signal at a different input signal power and launched pump power. The measured small-signal gain is above 14 dB from 1530 to 1565 nm. The obtained results show favorable performance of the RC-EDF-assisted OAM fiber amplifier in data-carrying OAM-DM and WDM systems.

## 2. Results

### 2.1. Concept and Principle

**Figure 1:**
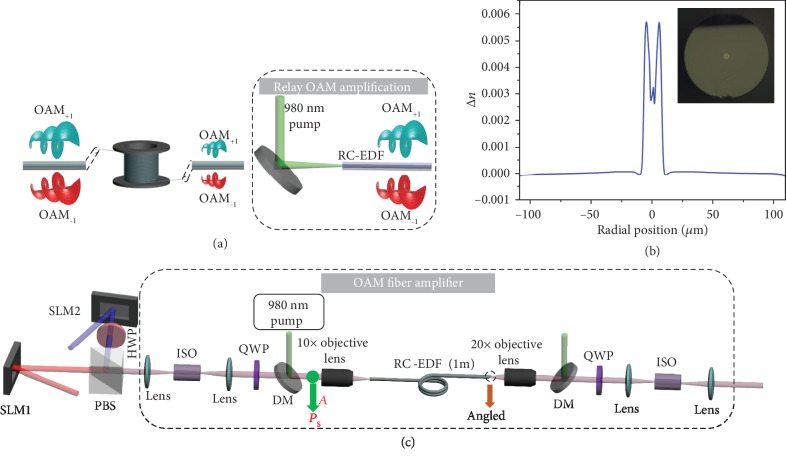
Conceptual illustration and experimental configuration of an OAM fiber amplifier based on a ring-core erbium-doped fiber (RC-EDF). (a) Concept and principle of the RC-EDF-assisted OAM fiber amplifier which provides relay amplification of multiplexed OAM modes after lossy long-haul fiber transmission. (b) Measured refractive index profile of the fabricated RC-EDF. The inset shows the measured optical microscope image of the fabricated RC-EDF facet. (c) Schematic illustration of the RC-EDF-assisted OAM fiber amplifier. SLM: spatial light modulator; PBS: polarization beam splitter; HWP: half-wave plate; ISO: isolator; QWP: quarter-wave plate; DM: dichroic mirror.

The concept and principle of an OAM fiber amplifier based on a RC-EDF are illustrated in Figure 1(a). The multiplexed OAM modes are greatly attenuated after long-haul fiber transmission and are difficult to be directly demultiplexed and detected. The OAM fiber amplifier provides the necessary relay amplification for multiplexed OAM modes after lossy long-haul fiber transmission. The OAM fiber amplifier is based on a RC-EDF. We design and fabricate a RC-EDF with its refractive index profile depicted in Figure 1(b). The insert image is the measured optical microscope image of the fabricated RC-EDF facet. The RC-EDF has an inner ring diameter of 4 *μ*m and outer ring diameter of 16 *μ*m and a cladding diameter of 219 *μ*m. The maximum refractive index difference between the ring core and silica cladding is 0.0057. The RC-EDF is designed to support OAM_+1_ and OAM_−1_ for an OAM-DM system.

The experimental configuration of the RC-EDF-assisted OAM fiber amplifier is schematically illustrated in Figure 1(c). OAM_−1_ and OAM_+1_ modes are generated by loading specific phase patterns onto two spatial light modulators (SLM1 and SLM2, respectively). The polarization of one OAM mode is rotated by 90° by a half-wave plate (HWP) after one SLM. Then, we use a polarization beam splitter (PBS) to combine the generated two linearly polarized OAM_−1_ and OAM_+1_ together. After that, the two OAM modes are converted into two circularly polarized OAM modes by the following quarter-wave plate (QWP). A 10x objective lens is used to couple the multiplexed OAM modes into a 1 m RC-EDF. Remarkably, to remove unwanted parasitic lasing, we cut obliquely at the fiber output. To reduce the potential Fresnel reflections and suppress the signal light propagation in the backward direction, we have two free-space isolators placed at both ends of the amplifier. A long-pass dichroic mirror (DMLP1180, Thorlabs, DM) is used to couple the 980 nm pump light into the RC-EDF, which is highly reflective below 1180 nm and highly transmissive above 1180 nm. To filter out residual pump beams, another DM is placed at the output. In addition, the input signal power (*P*_s_) is measured at point A.

### 2.2. Performance Evaluation of RC-EDF-Assisted OAM Fiber Amplifier

**Figure 2:**
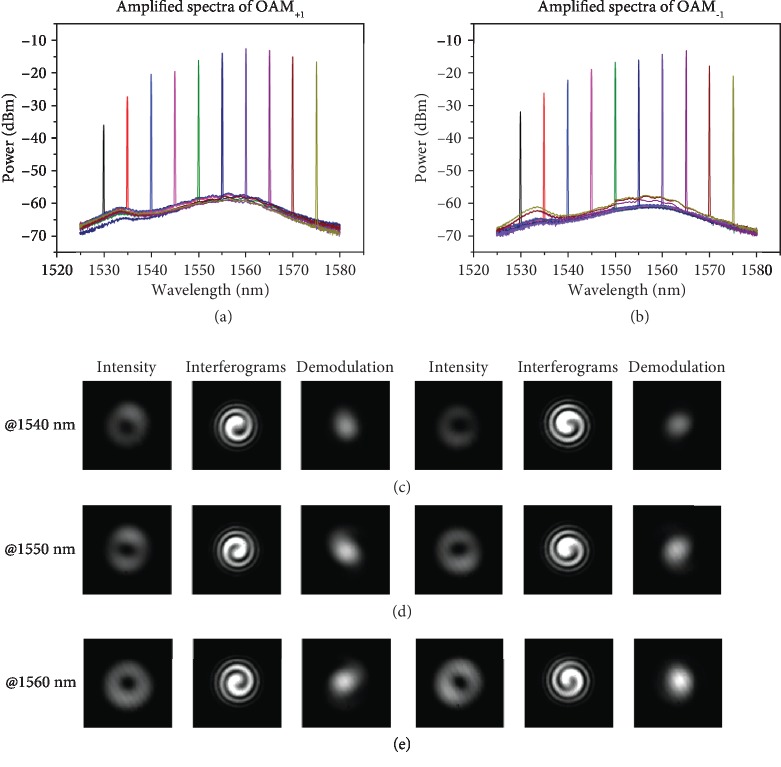
Measured amplified spectra, intensity distributions, interferograms, and demodulation of OAM modes for the RC-EDF-assisted OAM fiber amplifier. (a, b) Measured amplified spectra for OAM_+1_ (a) and OAM_−1_ (b) modes at the output of the RC-EDF-assisted OAM fiber amplifier. The pump power is 550 mW. (c–e) Measured intensity profiles, interferograms, and demodulation of OAM modes at wavelengths of 1540 (c), 1550 (d), and 1560 nm (e). Left: OAM_+1_ mode; Right: OAM_−1_ mode.

We first characterize the performance of the RC-EDF-assisted OAM fiber amplifier. Figures 2(a) and 2(b) show the measured amplified spectra for OAM_+1_ and OAM_−1_ modes of the RC-EDF-assisted OAM fiber amplifier at an input signal power of 5 dBm and launched pump power of 550 mW. It is shown that more than 30 dB optical signal-to-noise ratio (OSNR) after amplification with an OSA resolution of 0.1 nm can be achieved in the experiment. Figures 2(c)–2(e) display the measured intensity profiles, interferograms, and demodulation of OAM_+1_ and OAM_−1_ modes after amplification by the RC-EDF-assisted OAM fiber amplifier at the wavelengths of 1540, 1550, and 1560 nm, respectively. The left and right parts show the measured results for OAM_+1_ and OAM_−1_ modes, respectively, which can be verified by the twisting direction of the interferograms. It is noted that the interferograms are obtained by interfering OAM modes with a reference left-hand or right-hand circularly polarized Gaussian beam.

**Figure 3:**
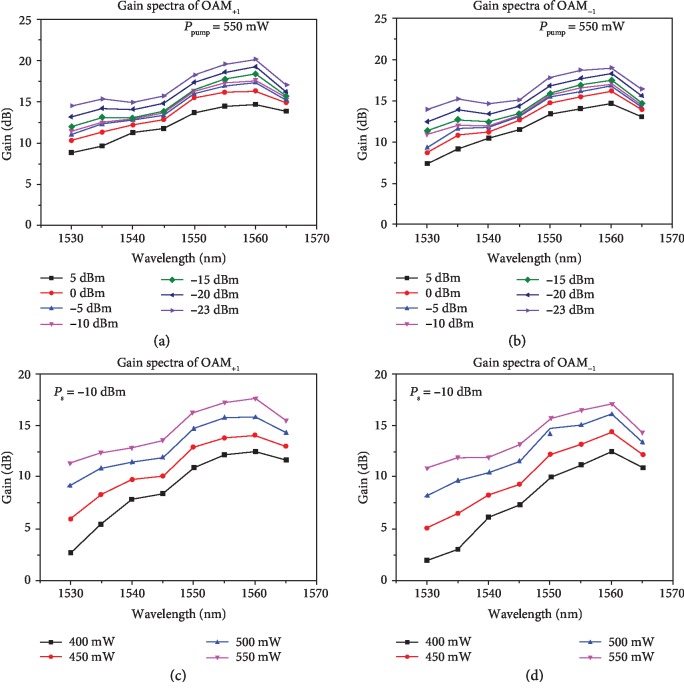
Measured gain spectra of the RC-EDF-assisted OAM fiber amplifier. Measured gain spectra for (a) OAM_+1_ and (b) OAM_−1_ modes versus wavelength with fixed pump power and (c) OAM_+1_ and (d) OAM_−1_ modes versus wavelength with fixed input signal power.

We then measure the gain spectra for OAM_+1_ and OAM_−1_ modes at the output of the RC-EDF-assisted OAM fiber amplifier as a function of the wavelength from 1530 to 1565 nm, as shown in Figures 3(a) and 3(b). The pump power (*P*_pump_) is fixed at 550 mW while *P*_s_ varies from -23 to 5 dBm. One can clearly see that the mode gain peak is located at 1560 nm with a maximum gain of 20.16 dB for the OAM_+1_ mode and 19.05 dB for the OAM_−1_ mode. In general, the mode gain reduces with the increase of the input signal power and the small-signal gain is up to 19 dB (*P*_s_ < ‐15 dBm). Similarly, we also measure the gain spectra for OAM_+1_ and OAM_−1_ modes as a function of the wavelength with *P*_pump_ increasing from 400 to 550 mW at a fixed *P*_s_ equaling -10 dBm, as shown in Figures 3(c) and 3(d). One can see that the mode gains of the OAM_+1_ and OAM_−1_ modes increase with the pump power. The mode gain peak is located at 1540 nm in the simulation while the experimental peak wavelength is 1560 nm. The slight differences between the simulations (see Materials and Methods) and experiments might be explained by the fact that the gain spectrum is highly associated with the signal and pump power and fiber length [37]. Additionally, the practical erbium-doping profile of the fabricated RC-EDF might be slightly different from the simulations adopting smooth interpolation curve calculated by five points.

### 2.3. Performance Evaluation of the Data-Carrying OAM-DM and WDM System Using RC-EDF-Assisted OAM Fiber Amplifier

**Figure 4:**
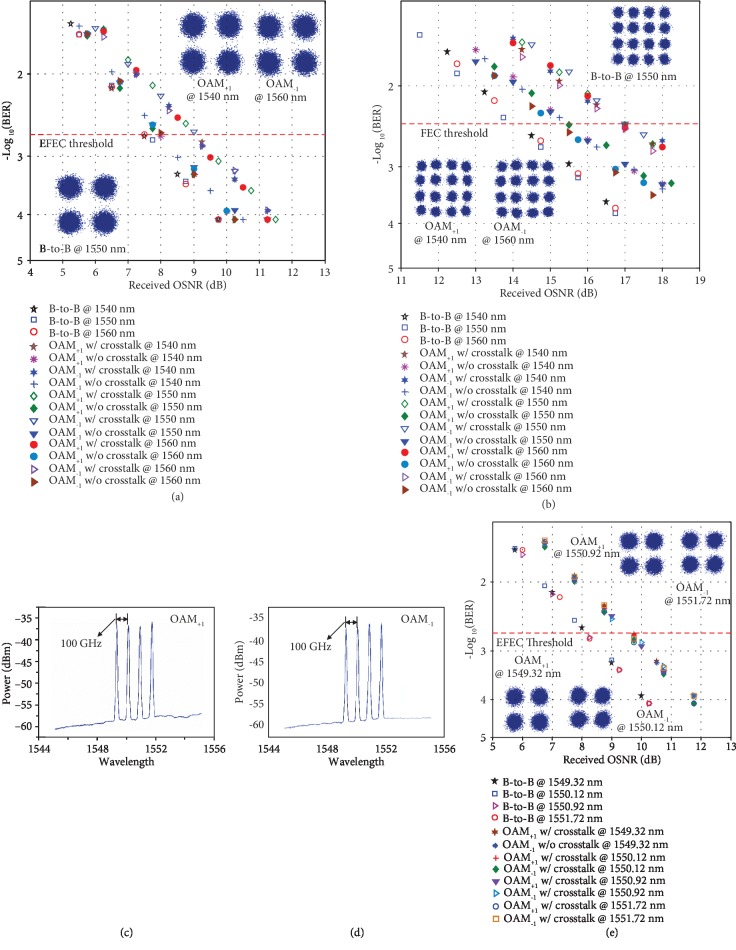
Measured BER performance of the data-carrying OAM-DM and WDM system using a RC-EDF-assisted OAM fiber amplifier. (a, b) Measured BER versus received OSNR of multiplexed OAM_+1_ and OAM_−1_ modes carrying 10-Gbaud QPSK (a) or 10-Gbaud 16-QAM (b) signal for single wavelength amplification at 1540, 1550, and 1560 nm, respectively. (c, d) Measured amplified spectra of OAM_+1_ (c) and OAM_−1_ (d) modes. (e) Measured bit-error rate (BER) versus received OSNR for amplification of multiplexed OAM_+1_ and OAM_−1_ modes over four 100 GHz ITU-grid wavelengths. EFEC: enhanced forward-error correction; w/o crosstalk: without crosstalk; w/ crosstalk: with crosstalk.

Furthermore, we apply the RC-EDF-assisted OAM fiber amplifier to the OAM-DM and WDM system and evaluate its system-level performance. The measured bit-error rate (BER) curves as a function of the received OSNR of the multiplexed OAM_+1_ and OAM_−1_ modes carrying 10-Gbaud QPSK or 16-QAM signal for the single wavelength at 1540, 1550, and 1560 nm are shown in Figures 4(a) and 4(b), respectively. The *P*_s_ is -15 dBm and the *P*_pump_ is 550 mW. The measured OAM_+1_- or OAM_−1_-only results without crosstalk (w/o crosstalk) are also depicted in Figure 4 for comparison with those with crosstalk (w/ crosstalk). For the 10-Gbaud QPSK signals, the measured OSNR penalties of demultiplexed OAM_+1_ and OAM_−1_ modes after amplification are about 0.5 dB w/o crosstalk and 1.5 dB w/ crosstalk at a BER of 2 × 10^−3^, the enhanced forward-error correction (EFEC) threshold, at the wavelength of 1540, 1550, and 1560 nm. The inserted images in Figure 4(a) depict measured typical constellations of 10-Gbaud QPSK signals for OAM_+1_ at 1540 nm, OAM_−1_ at 1550 nm, and OAM_+1_ at 1560 nm with crosstalk at a BER of ~1 × 10^−4^. For 10-Gbaud 16-QAM signals, the measured OSNR penalties of demultiplexed OAM_+1_ and OAM_−1_ modes after amplification are about 1.5 dB w/o crosstalk and 2.8 dB w/crosstalk at a BER of 3.8 × 10^−3^, the 7% hard-decision forward error correction (FEC) threshold, at the wavelength of 1540, 1550, and 1560 nm. The inserted images in Figure 4(b) show measured typical constellations of 10-Gbaud 16-QAM signals for OAM_−1_ at 1540 nm, OAM_+1_ at 1550 nm, and OAM_−1_ at 1560 nm with crosstalk at a BER of ~4 × 10^−3^. The obtained results indicate successful implementation of amplifying multiplexed data-carrying OAM modes with favorable system-level performance. Moreover, we measure the amplification performance of the data-carrying OAM-DM and WDM system. Figures 4(c) and 4(d) show the WDM spectra for OAM modes after amplification in which the four ITU-grid wavelengths are 1549.32 nm, 1550.12 nm, 1550.92 nm, and 1551.72 nm with a 100 GHz spacing. Figure 4(e) depicts the measured BER as a function of the received OSNR for multiplexed four wavelengths and two OAM modes carrying 10-Gbaud QPSK signals after amplification. The *P*_s_ is -15 dBm and the *P*_pump_ is 550 mW. The measured OSNR penalties of all the 8 channels (OAM-DM+WDM) are less than 1.8 dB at a BER of 2 × 10^−3^. The inserted images in Figure 4(e) are recorded constellations of 10-Gbaud QPSK signals for OAM_+1_ at 1549.32 nm and 1550.92 nm and OAM_−1_ at 1550.12 nm and 1551.72 nm with crosstalk at a BER of ~1 × 10^−4^. The obtained results indicate favorable performance of the data-carrying OAM-DM and WDM system using a RC-EDF-assisted OAM fiber amplifier.

**Figure 5:**
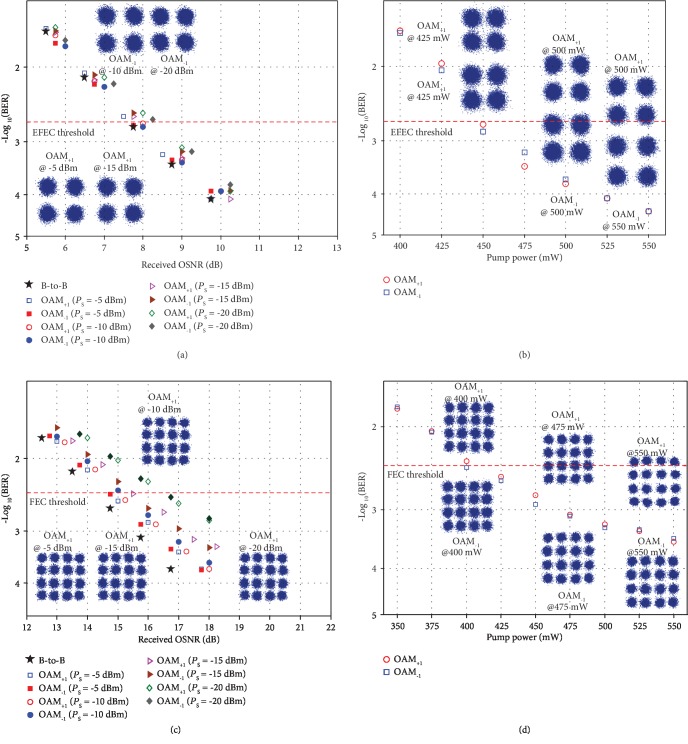
Measured BER performance of single OAM mode carrying a 10-Gbaud QPSK/16-QAM signal under different input signal power and launched pump power. (a, c) Measured BER versus received OSNR of single OAM_+1_ and OAM_−1_ modes carrying 10-Gbaud QPSK (a) and 16-QAM (c) signal under different input signal power with the pump power fixed at 550 mW and the signal wavelength at 1550 nm. (b, d) Measured BER versus launched pump power with the input signal power fixed at -15 dBm for OAM_+1_ and OAM_−1_ modes carrying 10-Gbaud QPSK (b) and 16-QAM (d) signal.

We also measure the BER performance of a single OAM mode carrying a 10-Gbaud QPSK/16-QAM signal at a different input signal power and launched pump power. Figures 5(a) and 5(c) display the measured BER performance for a single OAM_+1_ and OAM_−1_ mode carrying 10-Gbaud QPSK and 16-QAM signal at a different input signal power, respectively. The *P*_pump_ is 550 mW and the wavelength is 1550 nm. The overall BER performance is dependent on the input signal power fed into the RC-EDF-assisted OAM fiber amplifier which affects the output OSNR. The measured OSNR penalties of OAM_+1_ and OAM_−1_ modes are about 0.1, 0.3, 0.5, and 0.8 dB for the QPSK signal at a BER of 2 × 10^−3^ under different input signal powers of -5, -10, -15, and -20 dBm, respectively. The measured OSNR penalties of OAM_+1_ and OAM_−1_ modes are about 0.6, 1, 1.5, and 2.6 dB for the 16-QAM signal at a BER of 3.8 × 10^−3^ under a different input signal power of -5, -10, -15, and -20 dBm, respectively. The inserted images are typical constellations of QPSK and 16-QAM signals with a different input signal power. Moreover, we also measure the BER performance for OAM_+1_ and OAM_−1_ modes carrying a 10-Gbaud QPSK and 16-QAM signal at the output of the RC-EDF-assisted OAM fiber amplifier as a function of the launched pump power, as shown in Figures 5(b) and 5(d). The input signal power is fixed at -15 dBm. The inserted images depict measured typical constellations of QPSK and 16-QAM signals carried by OAM_+1_ and OAM_−1_ modes under a different pump power. One can clearly see that the BER performance is dependent on the pump power. Low pump power can lead to a rapid degradation in the BER performance.

The obtained results shown in Figures 2–5 indicate successful implementation of the RC-EDF-assisted OAM fiber amplifier for single and multiplexed OAM modes and its applications in a data-carrying OAM-DM and WDM system with favorable performance.

## 3. Discussion

In summary, we have proposed and demonstrated an RC-EDF-assisted OAM fiber amplifier, and its performance has been evaluated in a data-carrying OAM-DM and WDM system. The small-signal gain is up to 19 dB for both OAM_+1_ and OAM_−1_ modes at the wavelength of 1560 nm and above 14 dB from 1530 to 1565 nm (C band). When transmitting the 10-Gbaud QPSK signal, the measured OSNR penalties at a BER of 2 × 10^−3^ for single wavelength at 1540, 1550, and 1560 nm are less than 1.5 dB in an OAM-DM only system, while less than 1.8 dB for all channels in an OAM-DM and WDM system. When transmitting a 10-Gbaud 16-QAM signal, the measured OSNR penalties at a BER of 3.8 × 10^−3^ for a single wavelength at 1540, 1550, and 1560 nm are less than 2.8 dB in an OAM-DM only system. The overall BER performance is dependent on the input signal power and pump power fed into the OAM fiber amplifier. The obtained results indicate favorable performance of the OAM fiber amplifier based on RC-EDF. The demonstrated RC-EDF-assisted OAM fiber amplifier may open up added capacity scaling and long-haul transmission opportunities in high-speed optical communication systems employing OAM multiplexing. Remarkably, the OAM-DM is fully compatible with existing multiplexing techniques. In addition to the OAM-DM and WDM system, the RC-EDF-assisted OAM fiber amplifier can be also used in the OAM-DM system incorporating the polarization-division multiplexing (PDM) technique.

Beyond the proof-of-concept demonstration of the RC-EDF-assisted OAM amplifier in this work, it is essential to further improve the performance of the OAM fiber amplifier for practical use in long-haul OAM-DM communication systems. To further improve the performance of the RC-EDF, two issues need to be considered: (1) to support more OAM modes in the RC-EDF and (2) to enable large and uniform modal gain among different OAM modes, i.e., a specialty fiber providing large and uniform modal gain among more OAM modes is preferable. To increase the number of supported OAM modes, one way is to increase the refractive index difference between the fiber ring-core and cladding [16], i.e., high-index-contrast RC-EDF, and the other is to increase the ring width or position [35]. Air-core EDF might be also considered to further increase the refractive index difference between the ring core and cladding [36]. To enable large modal gain with low DMG over a wide wavelength range, the erbium-doping profile needs to be further optimized. For example, one can employ the RC-EDF design with two-layer erbium covering the sidewalls of the high-index region [38]. The newly added degree of freedom of the erbium-doping profile benefits the performance improvement. Besides, one can also employ a higher-order pump mode rather than the Gaussian beam to improve the overlap between the signal and pump intensity profiles for more uniform amplifications. Furthermore, in order to realize an all-fiber OAM-DM system which is the ultimate goal of optical fiber communication systems employing OAM modes, there are still many challenges to be addressed, such as all-fiber OAM (de)multiplexer, low-loss and low-crosstalk splicing of the EDF with the OAM transmission fiber, OAM fiber isolator, and all-fiber OAM 980/1550 nm WDM coupler. For example, splicing the ED-RCF with an OAM transmission fiber (e.g., vortex fiber [16, 34]) is of great importance. This could be addressed by precisely matching the mode profiles between the RC-EDF and OAM transmission fiber when designing the RC-EDF.

## 4. Materials and Methods

### 4.1. Simulation Results of RC-EDF

We theoretically assess the mode gain by solving the rate and power propagation equations through the Runge-Kutta 4^th^-order method [39, 40]. In this work, the simulations are under the following conditions: (1) The RC-EDF-assisted OAM fiber amplifier is assumed to be fundamentally pumped at the 980 nm wavelength, (2) OAM_+1_ and OAM_−1_ modes are considered, (3) the bandwidth of amplified spontaneous emission (ASE) ranges from 1430 to 1640 nm with an interval of 1 nm. We derive a new set of equations to describe the RC-EDF as follows:

(1) $$\begin{array}{c} \left\{\begin{array}{c} \frac{{dP}_{\text{pump}}\left(z\right)}{dz}=-P_{\text{pump}}\left(z\right)\int_{0}^{2\pi }\int_{0}^{a}\sigma_{\text{apump}}n_{1}\left(r,\phi ,z\right)i_{\text{pump}}\left(r,\phi \right)rdrd\phi ,\\ \frac{{dP}_{\text{signal},m}\left(z\right)}{dz}=P_{\text{signal},m}\left(z\right)\int_{0}^{2\pi }\int_{0}^{a}\left[\sigma_{\text{esignal}}n_{2}\left(r,\phi ,z\right)-\sigma_{\text{asignal}}n_{1}\left(r,\phi ,z\right)\right]i_{\text{signal},m}\left(r,\phi \right)rdrd\phi ,\\ \frac{{dP}_{{\text{ASE}}_{\lambda },m}\left(z\right)}{dz}=P_{{\text{ASE}}_{\lambda },m}\left(z\right)\int_{0}^{2\pi }\int_{0}^{a}\left[\sigma_{{\text{eASE}}_{\lambda }}n_{2}\left(r,\phi ,z\right)-\sigma_{{\text{aASE}}_{\lambda }}n_{1}\left(r,\phi ,z\right)\right]i_{{\text{ASE}}_{\lambda },m}\left(r,\phi \right)rdrd\phi ,+2h\nu_{{\text{ASE}}_{\lambda }}\Delta \nu_{{\text{ASE}}_{\lambda }}\int_{0}^{2\pi }\int_{0}^{a}\sigma_{{\text{eASE}}_{\lambda }}n_{2}\left(r,\phi ,z\right)i_{{\text{ASE}}_{\lambda },m}\left(r,\phi \right)rdrd\phi , \end{array}\right. \end{array}$$

(2) $$\begin{array}{c} \left\{\begin{array}{c} n_{1}\left(r,\phi ,z\right)=\frac{1/\tau +W_{3}\left(r,\phi ,z\right)}{1/\tau +W_{1}\left(r,\phi ,z\right)+W_{2}\left(r,\phi ,z\right)+W_{3}\left(r,\phi ,z\right)}\rho \left(r,\phi \right),\\ n_{2}\left(r,\phi ,z\right)=\frac{W_{1}\left(r,\phi ,z\right)+W_{2}\left(r,\phi ,z\right)}{1/\tau +W_{1}\left(r,\phi ,z\right)+W_{2}\left(r,\phi ,z\right)+W_{3}\left(r,\phi ,z\right)}\rho \left(r,\phi \right),\\ n_{1}\left(r,\phi ,z\right)+n_{2}\left(r,\phi ,z\right)=\rho \left(r,\phi \right), \end{array}\right. \end{array}$$

(3) $$\begin{array}{c} \left\{\begin{array}{c} W_{1}\left(r,\phi ,z\right)=\frac{\sigma_{\text{apump}}P_{\text{pump}}i_{\text{pump}}\left(r,\phi ,z\right)}{h\nu_{\text{pump}}},\\ W_{2}\left(r,\phi ,z\right)=\underset{m}{\sum }\frac{\sigma_{\text{asignal}}P_{\text{signal},m}i_{\text{signal},m}\left(r,\phi ,z\right)}{h\nu_{\text{signal}}}+\underset{m}{\sum }\underset{\lambda }{\sum }\frac{\sigma_{{\text{aASE}}_{\lambda }}P_{{\text{ASE}}_{\lambda },m}\;i_{{\text{ASE}}_{\lambda },m}\left(r,\phi ,z\right)}{h\nu_{{\text{ASE}}_{\lambda }}},\\ W_{3}\left(r,\phi ,z\right)=\underset{m}{\sum }\frac{\sigma_{\text{esignal}}P_{\text{signal},m}i_{\text{signal},m}\left(r,\phi ,z\right)}{h\nu_{\text{signal}}}+\underset{m}{\sum }\underset{\lambda }{\sum }\frac{\sigma_{{\text{eASE}}_{\lambda }}P_{{\text{ASE}}_{\lambda },m}\;i_{{\text{ASE}}_{\lambda },m}\left(r,\phi ,z\right)}{h\nu_{{\text{ASE}}_{\lambda }}}. \end{array}\right. \end{array}$$

In equations (1)–(3), *m* indicates different modes, *σ*_a_ and *σ*_e_ are the absorption and emission cross-sections, respectively; *n*_1_, *n*_2_, and *ρ* are the erbium ion densities of the ground state, the metastable state, and the total erbium ion density; variables *υ* and Δ*υ* designate the optical frequency and the ASE channel bandwidth centered at *υ*; *h* is the Planck constant; and *τ* is the lifetime of the metastable level of the erbium ions. *i*(*r*, *ϕ*) is the normalized beam intensity profile calculated by using a full-vector finite-element mode solver. For the OAM_+1_ and OAM_−1_ modes, *i*_signal_(*r*, *ϕ*) is obtained by combining the fiber eigenmodes in the form of HE_21_^even^ ± *i*∗HE_21_^odd^. By solving equations (1)–(3) through the Runge-Kutta 4^th^-order calculation, the gain for OAM_+1_ and OAM_−1_ modes of RC-EDF can be assessed.

**Figure 6:**
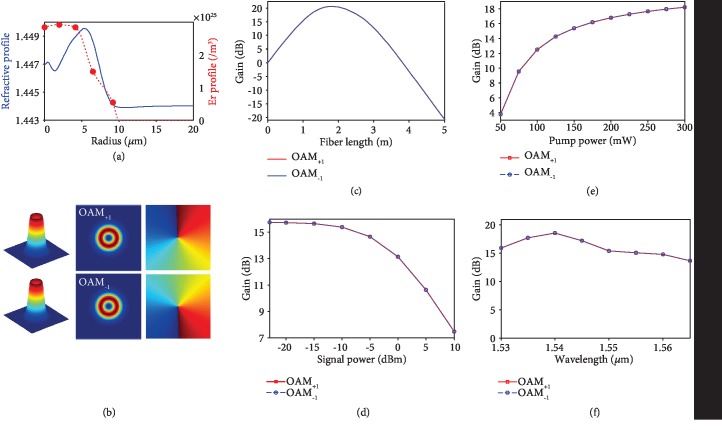
Simulation results of the RC-EDF-assisted OAM fiber amplifier. (a) Measured refractive index profile and erbium-doping profile of the fabricated RC-EDF. (b) Simulated 3D intensity profiles, 2D intensity profiles, and phase profiles at the cross-section of OAM_+1_ and OAM_−1_ modes. (c–f) Simulated mode gain for OAM_+1_ and OAM_−1_ modes versus fiber length (c), signal power (d), pump power (e), and wavelength (f).

The measured refractive index profile and erbium-doping profile of the fabricated RC-EDF are plotted in Figure 6(a). The index profile of Figure 6(a) is plotted with the different scale from Figure 1(b) in the radius, which depicts the ring-core structure more clearly. The five red solid circles are the measured erbium ion doping concentration in five radial positions, and the red dashed line is the related smooth interpolation curve for simulation. The simulated results of OAM_+1_ and OAM_−1_ modes supported in the fabricated RC-EDF are shown in Figure 6(b) in the upper and lower rows, respectively. The 3D intensity profiles, 2D intensity profiles, and phase profiles at the cross-section of OAM_+1_ and OAM_−1_ modes are displayed in the first column, second column, and third column, respectively. One can clearly see the doughnut intensity profiles due to the phase singularity at the beam center.

Figures 6(c)–6(f) plot the simulated mode gain of OAM_+1_ and OAM_−1_ modes based on the smooth interpolation curve of the erbium-doping profile shown in Figure 6(a). The parameters of the RC-EDF-assisted OAM fiber amplifier used in simulations are as follows. The fiber length is 1 m, pump power is 150 mW, signal power is -10 dBm, and the signal wavelength is 1550 nm. The gain is simulated by scanning one of the parameters with the others fixed as above. For example, Figure 6(c) shows the gain for OAM_+1_ and OAM_−1_ modes versus fiber length with pump power, signal power, and wavelength fixed at 150 mW, -10 dBm, and 1550 nm, respectively. The gain for the OAM_+1_ and OAM_−1_ modes is 15.38 dB with a fiber length of 1 m. Similarly, when fixing the fiber length at 1 m, the gains for OAM_+1_ and OAM_−1_ modes versus signal power, pump power, and wavelength are shown in Figures 6(d)–6(f), respectively. One can see that the gain increases with the pump power, while the growth rate becomes relatively slow with larger pump power. The smaller signal power benefits larger gain. The gain peak is located at ~1540 nm with the maximum gain of 18.54 dB for the OAM_+1_ and OAM_−1_ modes over the whole C band.

### 4.2. Experimental Realization

**Figure 7:**
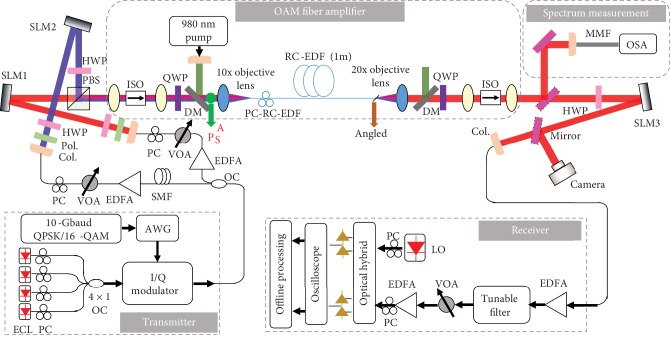
Experimental setup of the RC-EDF-assisted OAM fiber amplifier in OAM-DM and WDM systems. The setup consists of five parts (transmitter, multiplexing of OAM modes, OAM fiber amplifier based on RC-EDF, demultiplexing of OAM modes, and receiver). ECL: external cavity laser; PC: polarization controller; I/Q modulator: in-phase/quadrature modulator; OC: optical coupler; Col.: collimator; Pol.: polarizer; PC-RC-EDF: polarization controller on RC-EDF; MMF: multimode fiber; LO: local oscillator; OSA: optical spectrum analyzer.

The detailed experimental setup of the RC-EDF-assisted OAM fiber amplifier in data-carrying OAM-DM and WDM systems is shown in Figure 7, which consists of five parts (transmitter, multiplexing of OAM modes, OAM fiber amplifier based on RC-EDF, demultiplexing of OAM modes, and receiver). At the transmitter side, a 10-Gbaud QPSK or 16-QAM signal is generated by the tunable laser followed by an optical I/Q modulator, which is fed by an arbitrary waveform generator (AWG, Tektronix AWG70002) with programmed 10-Gbaud QPSK/16-QAM electric signal. A laser at a wavelength of 1540, 1550, or 1560 nm is used as the optical source for the single wavelength system. Four lasers at wavelengths of 1549.32, 1550.12, 1550.92, and 1551.72 nm are employed as optical sources for a typical 100 GHz ITU-grid WDM system. In the multiplexing part, before incident to the SLMs, the light beam is split into two paths, relatively delayed by a piece of SMF for decorrelation, and amplified by erbium-doped fiber amplifiers (EDFAs). The following part is an OAM fiber amplifier assisted by a RC-EDF, which is described in Figure 1(c). A polarization controller on RC-EDF (PC-RC-EDF) is used to mitigate the mode crosstalk. After being amplified by the RC-EDF-assisted OAM fiber amplifier, the OAM modes are collected by a 20x objective lens and converted back to a Gaussian-like beam by another SLM loaded with a specific phase pattern. A camera is used in the multiplexing and demultiplexing parts to observe the intensity distributions of output light beams of the OAM fiber amplifier and demodulated OAM modes.

At the receiver side (coherent detection), the back-converted Gaussian-like beam with a bright spot at the beam center is coupled into an SMF, amplified by a second EDFA. A tunable filter is used to suppress the ASE noise. A variable optical attenuator (VOA) followed by a third EDFA are then used to adjust the received OSNR for BER performance measurement. Another tunable laser serves as a local oscillator (LO) to mix with the received signal by an optical hybrid, the outputs of which are sent to an oscilloscope followed by offline DSP. To characterize the performance of the OAM fiber amplifier, an additional part is set for measuring amplified spectra of the RC-EDF-assisted OAM fiber amplifier before the demultiplexing part. The input signal power (*P*_s_) is measured by a free-space power meter (PM100D, S132C) at the point A before the 10x objective lens. The output light beams after amplification are collected by a MMF and fed into the optical spectrum analyzer (OSA, Yokogawa AQ6370C). To eliminate effects of the noise fluctuation with the input signal power and pump power, the output power (*P*_out_) is measured by an OSA at the range of 10 nm around the input signal wavelength. Considering the loss of optical elements (objective lens, lens, isolator, QWP, and DM) and the coupling loss of RC-EDF (*P*_loss_), the gain of the OAM fiber amplifier can be indicated as Gain = *P*_out_ + *P*_loss_ − *P*_s_. By scanning the input wavelength at an interval of 5 nm, we can get the gain at different wavelengths and then plot the amplified spectra of the RC-EDF-assisted OAM fiber amplifier.
